# RECRUITING AND RETAINING RESIDENTIAL CARE COMMUNITIES DURING THE COVID-19 PANDEMIC: LESSONS LEARNED

**DOI:** 10.1093/geroni/igad104.2586

**Published:** 2023-12-21

**Authors:** Silvia Orsulic-Jeras, Ashlee Cordell, Sara Powers, Farida Ejaz, Lisbeth Sanders

**Affiliations:** Benjamin Rose Institute on Aging, Cleveland, Ohio, United States; Benjamin Rose Institute on Aging, Cleveland, Ohio, United States; Benjamin Rose Institute on Aging, Cleveland, Ohio, United States; Benjamin Rose Institute on Aging, Cleveland, Ohio, United States; LifeBio, Marysville, Ohio, United States

## Abstract

Demands within residential care communities have escalated throughout the Covid-19 pandemic as meeting basic care needs has become more critical. Non-pharmacological interventions that aim to alleviate care strain and promote person-centered care are even more important now, as residents have been significantly impacted by social isolation and loss. This presentation highlights successes and challenges faced by researchers and residential care communities related to study recruitment and retainment efforts during the Covid-19 pandemic while conducting a fidelity trial of LifeBio MemoryTM: a novel app-based product designed to collect life story information from persons living with dementia (PLWD). The target sample is comprised of residential care staff (n=60) and PLWD (n=242) across ten residential care communities in the state of Ohio. Successes presented include: 1) high enthusiasm for the study and 2) strong relationships built between researchers and site liaisons. Significant challenges faced include: 1) organization-wide staff shortages, 2) low overall resident census, and 3) Covid-19 outbreaks and quarantines. Key lessons learned include: 1) researchers and residential care communities face competing demands as a result of the Covid-19 pandemic and 2) the Covid-19 pandemic has changed the landscape of residential care and research. The importance of efficient field operations, supportive training resources, and contingency plans when conducting studies in residential care will be discussed.

